# Thermal conductivity of highly porous Si in the temperature range 4.2 to 20 K

**DOI:** 10.1186/1556-276X-9-318

**Published:** 2014-06-25

**Authors:** Katerina Valalaki, Androula Galiouna Nassiopoulou

**Affiliations:** 1 NCSR Demokritos/INN, Terma Patriarchou Grigoriou, Aghia Paraskevi, Athens 15310, Greece

**Keywords:** Porous Si, Thermal conductivity, Cryogenic temperatures, Nanoscale semiconductors

## Abstract

We report on experimental results of the thermal conductivity *k* of highly porous Si in the temperature range 4.2 to 20 K, obtained using the direct current (dc) method combined with thermal finite element simulations. The reported results are the first in the literature for this temperature range. It was found that porous Si thermal conductivity at these temperatures shows a plateau-like temperature dependence similar to that obtained in glasses, with a constant *k* value as low as 0.04 W/m.K. This behavior is attributed to the presence of a majority of non-propagating vibrational modes, resulting from the nanoscale fractal structure of the material. By examining the fractal geometry of porous Si and its fractal dimensionality, which was smaller than two for the specific porous Si material used, we propose that a band of fractons (the localized vibrational excitations of a fractal lattice) is responsible for the observed plateau. The above results complement previous results by the authors in the temperature range 20 to 350 K. In this temperature range, a monotonic increase of *k* with temperature is observed, fitted with simplified classical models. The extremely low thermal conductivity of porous Si, especially at cryogenic temperatures, makes this material an excellent substrate for Si-integrated microcooling devices (micro-coldplate).

**PACS:**

61.43.-j; 63.22.-m; 65.8.-g

## Background

Highly porous Si is a material composed of interconnected Si nanowires and nanocrystals separated by voids [1,2]. Due to its structure and morphology, it shows much lower thermal conductivity than that of bulk crystalline Si, which is even below the amorphous limit at porosities exceeding 60%. This is attributed to phonon confinement in the Si nanostructures and phonon scattering at porous Si large internal surface. The room temperature thermal conductivity of porous Si was extensively investigated in the literature (see a list in [3]), and the material is now established as an effective low thermal conductivity substrate for Si-based thermal devices [4], including flow sensors [5-8], gas sensors [9], accelerometers [10], and thermoelectric devices [11,12]. An increasing interest is recently devoted to the potential use of porous Si as a thermoelectric material with high figure of merit (ZT), achievable with its low thermal conductivity, combined with an intentional doping to increase its electrical conductivity [13-15].

In spite of the extensive literature related to the room temperature thermal conductivity of porous Si, only few works were devoted to its temperature dependence, and especially at cryogenic temperatures [16-18]. These measurements are essential for the assessment of the use of this material as a substrate for Si-based cooling devices (micro-coldplates). De Boor et al. [16] measured the thermal conductivity of porous silicon formed on n-type silicon in the temperature range 120 to 450 K using the 3*ω* method. Gesele et al. [17] used the same method to measure the thermal conductivity of porous silicon from both p and p^+^-type silicon in the temperature range 35 to 350 K. In a most recent paper by the authors of this paper [18], the thermal conductivity of mesoporous Si from p-type Si wafers with resistivity in the range 1 to 10 Ω cm, and 63% porosity was measured for temperatures from 20 to 350 K. The above material was nanostructured with randomly distributed pores in a sponge-like morphology. It was found that the temperature dependence of the thermal conductivity of this type of porous Si in the above temperature range is monotonic and does not show any maximum, as in the case of bulk crystalline Si and other crystalline materials. It is more similar to that of different low thermal conductivity amorphous materials, its value being even lower than that of the most known such materials (amorphous Si, silicon oxide, silicon nitride). The thermal conductivity of highly porous Si at cryogenic temperatures is more than four orders of magnitude lower than that of bulk crystalline Si [18]. This is mainly due to its porous nanoscale structure that causes phonon confinement and phonon-wall scattering that blocks thermal transport [19,20].

In this study, we extend previous measurements of the temperature dependence of porous Si thermal conductivity to the low temperature range 4.2 to 20 K. We found that at these low temperatures, porous Si thermal conductivity is almost stable with temperature, showing a plateau-like behavior. This behavior is common to glasses and disordered materials (i.e., SiO_2_, vitreous silica, epoxy resin, etc.), but unusual in crystalline systems. The plateau-like behavior of porous Si thermal conductivity in the above temperature range will be discussed by considering the fractal nature of the material and the existence of localized vibrational excitations (fractons) that dominate at these temperatures. At higher temperatures, other mechanisms are dominant and will be discussed.

The obtained absolute values of thermal conductivity of the studied nanostructured porous Si are lower than those of many known low-k materials in the whole temperature range 5 to 350 K. This demonstrates the high potential of this material as a substrate for thermal isolation on the Si wafer (micro-hotplate or micro-coldplate for Si-based thermal and cooling devices).

## Methods

Highly porous Si layers, 40 μm thick, were locally formed on a p-type (100) Si wafer with resistivity 1 to 10 Ω cm by anodization in a hydrofluoric acid (HF)/ethanol solution under a constant current density of 80 mA/cm^2^. The material porosity was 63% and was verified by using the well-known three-weight measurement method. The average pore diameter was 6 nm (mesoporous material).

**Figure 1:**
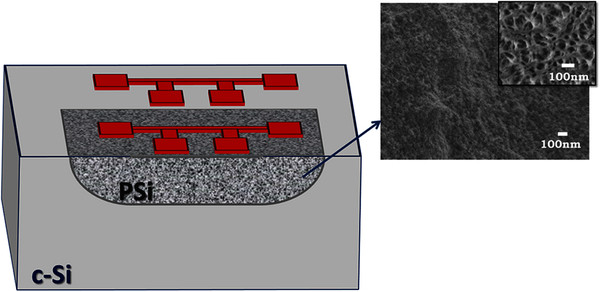
**Schematic representation of the test structure.** The figure shows a schematic representation of the locally formed porous Si layer on the p-type wafer and SEM images of the porous Si surface. The SEM image in the inset of the principal one was obtained after a slight plasma etching of the porous Si surface in order to better reveal the porous structure. Two resistors, one on porous Si and one on bulk Si, are also depicted in the schematic of the test structure.

The steady-state direct current (dc) method, described in detail in [18] and [21], was used to determine porous Si thermal conductivity. This method is based on the measurement of the temperature difference across a Pt resistor lying on the porous Si layer in response to an applied heating power. A similar resistor on bulk crystalline Si served as a temperature reference. Figure  1 shows schematically the locally formed porous Si layer with the Pt resistor on top, while the second resistor on bulk Si is also depicted. Scanning electron microscopy (SEM) images of the specific porous Si material are also depicted in the same figure. The SEM image in the inset was obtained after a slight plasma etching of the porous Si surface in order to better reveal the porous Si structure.

## Results and discussion

**Figure 2:**
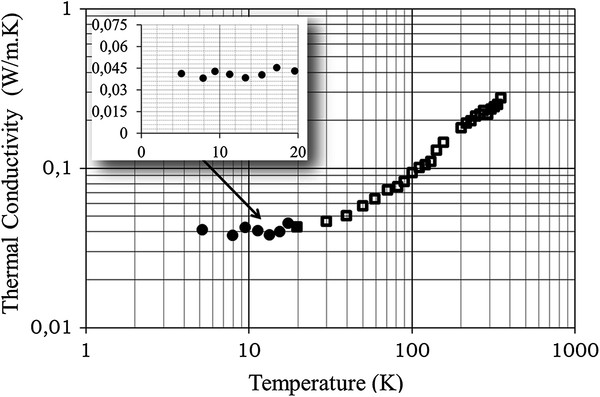
**Temperature dependence of porous Si thermal conductivity.** The graph shows experimental results of thermal conductivity of porous Si for temperatures between 5 and 20 K (present results, full points in the main figure and in the inset) and for temperatures in the range 20 to 350 K (open rectangles; previous results by the authors [18]). The plateau-like behavior for the 5 to 20 K temperature range is illustrated, with a mean value of 0.04 W/m.K.

For the extraction of the substrate thermal conductivity, a combination of experimental results and finite element method (FEM) analysis was used. The obtained results in the temperature range 5 to 20 K are depicted by full black circles in Figure  2 and in the inset of this figure. Plateau-like temperature dependence at a mean value of approximately 0.04 W/m.K was obtained. These results are the first in the literature in the 5 to 20 K temperature range. For the sake of completeness, our previous results for temperatures between 20 and 350 K are also presented in the same figure by open rectangles. A monotonic increase of the thermal conductivity as a function of temperature is obtained for temperatures above 20 K and up to 350 K, without any maximum as that obtained, in the case of bulk crystalline Si.

Plateau-like behavior of the temperature dependence of thermal conductivity at low temperatures is commonly observed in glasses and disordered materials and is attributed to different mechanisms [22], including phonon scattering by enhanced densities of tunnelling systems, elastic scattering by density fluctuations, dimensional crossover of the vibrational density of states, phonon localization, and fractons. Porous Si material is also characterized by disorder and has been described by several authors as a fractal network with specific fractal geometry. The fractal networks were extensively studied in the literature to understand the thermodynamics and transport properties of random physical systems. In [23] and [24], the authors considered the dynamics of a percolating network and developed a fundamental model for describing geometrical features of random systems. By taking a self-similar fractal structure, they evaluated the density of states for vibrations of a percolation network with the introduction of the fracton dimension $\tilde{d}$:

(1) $$\tilde{d}=\frac{2\times \bar{d}}{\left(2+\theta \right)}$$

where $\bar{d}$ is the so-called Hausdorff dimensionality and *θ* is a positive exponent giving the dependence of the diffusion constant on the distance. More details about the problem of fracton excitations in fractal structures, and generally the dynamical properties of fractal networks, are found in [25].

Rammal and Toulouse [23] showed that fractons are spatially localized vibrational excitations of a fractal lattice, obtained in materials with fracton dimension $\tilde{d}<2$.

In general, fractal geometry is observed in porous materials. Several works were devoted to the investigation of the fractal geometry of porous Si [26,27] and the use of the fractal nature of this material to explain its different physical properties, as for example its alternating current (ac) electrical conductivity [26]. Porous Si constitutes an interesting system for the study of fundamental properties of disordered nanostructures. There are no grain boundaries as in crystalline solids and no sizable bond angle distortions as those found in disordered non-crystalline systems, e.g., in amorphous materials. Porous silicon is thus considered as a simple mathematical ‘percolation’ model system, which is created by randomly removing material from a homogeneous structure, but still maintaining a network between the remaining atoms. Percolation theory has been recently used in the literature to describe thermal conduction in porous silicon nanostructures [28], amorphous and crystalline Si nanoclusters [29], nanotube composites [30], and other materials.

**Figure 3:**
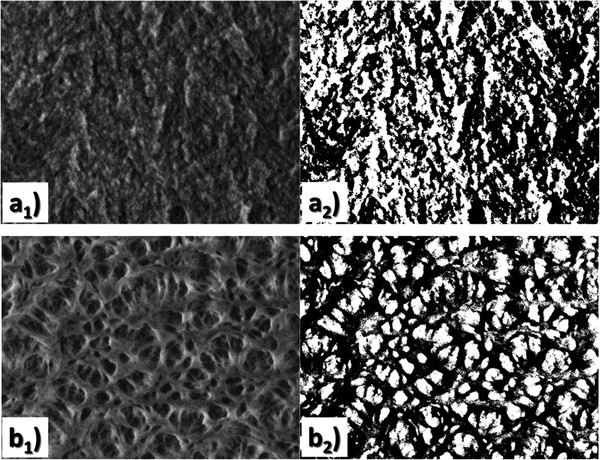
**Porous Si SEM images used for the calculation of Hausdorff dimension.** Examples of cross-sectional SEM images **(a**_**1**_**)** and top view images **(b**_**1**_**)** of the studied porous Si layer with their corresponding binary images **(a**_**2**_**)** and **(b**_**2**_**)**, used for the calculation of the box counting dimension.

We derived the Hausdorff dimension $\bar{d}$ of our porous Si material using scanning electron microscopy (SEM) images and the box counting algorithm [31]. The SEM images reflect the fractal microstructure of the material. The box counting dimension is then defined, which is a type of fractal dimension and is based on the calculation of a scaling rule (using the negative limit of the ratio of the log of the number of boxes at a certain scale over the log of that scale). The open-access software ‘ImageJ’ [32] was used for the SEM image processing, while the open-access software ‘FracLac’ [33] was used to calculate the Hausdorff dimension of our SEM images using the standard non-overlapping box counting method. We used the maximum possible different grid positions for every image in order to ensure the accuracy of the calculation, while we calculated the box counting dimension for both cross-sectional and top view SEM images of different magnifications. The results were similar from both top-view and cross-sectional images. We also used SEM images from different samples that were prepared with the same electrochemical conditions. In all cases, the calculated Hausdorff dimension was found to be less than two, including the standard error. Some examples of the images used and their corresponding binary ones are shown in Figure  3. The average of $\bar{d}$ values was approximately 1.822 ± 0.084. Since $\bar{d}$ is less than two, it is evident from expression (1) that $\bar{d}$ is also lower than two, since *θ* is a positive quantity. The condition for the existence of fractons in our system is thus fulfilled.

From the above, it results that our specific porous Si material used in this work shows Hausdorff dimensionality smaller than 2 and consequently (see above) a fracton dimension also smaller than 2. This last condition is considered as a necessary condition for the existence of fractons in the material. The observed plateau-like behavior of porous Si thermal conductivity at temperatures in the range 5 to 20 K can thus be attributed to the dominance of fractons, as in the case of other disordered materials [34,35].

The fracton formalism is also supported by the existence of the so-called ‘Boson peak’ in the Raman spectra and by the Brillouin spectra of porous Si, observed by different groups in the literature. The Boson peak is considered as a signature of the existence of localized vibrational modes in amorphous materials. For example, Shintani and Tanaka [36] correlated the Boson peak for glasses with the Ioffe-Regel frequency, which is the frequency reached when the mean free path for phonons approaches their wavelength and is a limit above which transverse phonon modes no longer propagate [37]. Foret et al. [38] investigated acoustic localization in fused silica and claimed that the states near the Boson peak are localized and satisfy the Ioffe-Regel criterion. In a fractal geometry, the non-propagating phonon modes are called fractons [24]. Therefore, in a fractal geometry, there is also a link between the appearance of a Boson peak in the Raman spectra and the existence of fractons.

Low-frequency Raman modes of nanometric Si crystallites were first observed in porous Si [39,40]. Gregora et al. [39] observed a well-defined peak at 37 cm^-1^ in the low-frequency spectra of nanostructured porous silicon with 70% porosity. Confined acoustic phonons were also observed in Si nanocrystals, of diameter 3.1 nm, dispersed in SiO_2_[41], and a broad peak between 20 and 40 cm^-1^ was observed both in polarized and depolarized spectra, which could be attributed to a Boson peak, even though the authors did not explicitly name it as such. In addition, the Raman spectrum of porous silicon studied in [42] revealed a Boson peak at 150 cm^-1^. In a recent work, Claudio et al. [43] observed a Raman peak at 6 meV (approximately 50 cm^-1^) in doped polysilicon nanoparticles that were exposed to air and sintered to form nanocrystalline silicon. Their material had similar structure to that of our studied porous Si layer. They attributed the observed peak to a Boson peak.

Brillouin spectroscopy is also a method to study the different phonon modes of a material. By applying it to porous Si with 80% porosity, Lockwood et al. [44] identified two acoustic phonon peaks exhibiting large peak widths. They attributed these peaks to the existence of fractons. However, in a more recent work of the same authors [45], the peak at 8 GHz was absent from their Brillouin spectra. The peak at 14 GHz observed by Lockwood was also observed by them, but it was attributed by the authors to the bulk transverse Rayleigh mode. In a recent paper by Polomska-Harlick and Andrews [46], a peak at approximately 8 GHz was observed in the Brillouin spectrum of porous Si with 59% porosity, similar to that observed by Lockwood et al. [44]. Even though the authors characterized this peak as ‘unknown’, we think that it could be attributed to the existence of the phonon-to-fracton crossover, suggested by Lockwood for porous Si and also observed in other disordered materials [35]. Its intensity increased with sin *θ* and saturated at sin *θ* ~ 0.9 ⇒ *θ* ~ 65°. Based on the above two references, if we consider the Brillouin peak frequency at approximately 8 GHz as the crossover frequency, *f*_co_, a crossover temperature *T*_co_ ~ 0.4 K is calculated.

In amorphous materials, the high temperature limit of the plateau is at around 20 K. Above the plateau, a linear increase of the thermal conductivity with increasing temperature is observed. Alexander et al. [47] introduced the anharmonic interaction between fractons and phonons in order to explain this linear increase. While fractons do not carry heat, and as a result their existence leads to a constant value of thermal conductivity with temperature, through the fracton-phonon interaction phonon-induced fracton hopping can contribute to the heat current, generating a thermal conductivity which increases linearly with increasing temperature.

Our porous Si thermal conductivity results show a plateau in the temperature range 5 to 20 K, with a constant value of 0.04 W/m.K, and a monotonic increase of the thermal conductivity with temperature, at temperatures above 20 K. In the temperature range 30 to 100 K, we observed an almost linear temperature dependence of the thermal conductivity, as that discussed by Alexander et al. [47] for amorphous materials. For higher temperatures, the temperature dependence deviates from linearity and fractons cannot be considered as the dominant mechanism. Our experimental results for highly porous Si at temperatures higher than 100 K [18] were fitted by models considering a simplified porous Si structure, as for example the phonon diffusion model by Gesele et al. [17] and the phonon hydrodynamic model by Alvarez et al. [48]. A comparison of our experimental results with the above models was made in [18]. Very good agreement with the phonon diffusion model was obtained for temperatures in the range 200 to 350 K, while a better qualitative description of the temperature dependence of *k* in a larger temperature range (100 to 350 K) was obtained with the phonon hydrodynamic approach. We have to note here that discrepancies between the experimental results and the different theoretical models as the ones above are mainly due to the very complicated structure of porous Si, which is not fully taken into account by the models. Nanostructured porous Si is composed of interconnected Si nanowires and nanocrystals, covered by a native oxide shell and separated by voids. The ratio of the native oxide compared to the Si core plays a critical role in the determination of the mechanism of thermal conduction in the different temperature ranges, especially at cryogenic temperatures [49]. This is because of the different temperature dependence of vibrational modes in the two systems (the Si backbone and the shell oxide).

## Conclusions

The thermal conductivity of 63% porosity nanostructured porous Si was measured for the first time in the cryogenic temperature range 5 to 20 K. A stable value as low as 0.04 W/m.K was obtained in this temperature range. We attribute the plateau-like behavior of our porous Si material at cryogenic temperatures to the presence of fractons, which are localized anomalous vibrational modes according to the scaling theory of localization of Rammal and Toulouse. We discussed in detail the specific fractal geometry of our porous Si system and its fractal dimensionality that supports the adoption of the fracton formalism. Literature results demonstrated the existence of the so-called Boson peak in the micro-Raman spectra of porous Si with a similar porosity than that of the porous Si layer used in this work. The existence of this peak in a material is in general considered as a signature of the presence of localized vibrational modes (‘fractons’ in a fractal lattice). In addition, literature results of Brillouin spectra of porous Si also showed localized vibrational modes that support our interpretation. Above the plateau and up to approximately 100 K, an almost linear increase with temperature was obtained for our highly porous Si material, as that obtained in amorphous materials and attributed to the anharmonic interaction between fractons and phonons. Above 100 K, other mechanisms are dominant, introduced into different models, like the phonon diffusion model that describes nicely well our results in the temperature range 200 to 350 K.

## Competing interests

The authors declare that they have no competing interests.

## Authors' contributions

KV made the experiments and wrote a first draft of the manuscript while AGN supervised the work and fully revised the paper. Both authors read and approved the final manuscript.
